# An Exceptional Preaortic Ectopic Type B2 Thymoma as the Cause of Refractory Myasthenia Gravis: A Case Report

**DOI:** 10.7759/cureus.111502

**Published:** 2026-06-25

**Authors:** Douaa Elmejdoubi, Oumayma Azzou, Yasmina Zakaria, Imane Zouidine, Nissrine Louhab

**Affiliations:** 1 Neurology, Faculty of Medicine and Pharmacy, Cadi Ayyad University, Mohammed VI University Hospital, Marrakech, MAR; 2 Radiology, Mohammed VI University Hospital, Marrakech, MAR

**Keywords:** aortic region thymoma, ectopic thymoma, myasthenia gravis, refractory myasthenia gravis, thymoma type b2

## Abstract

Myasthenia gravis (MG) is a chronic autoimmune neuromuscular disorder mediated by autoantibodies directed against acetylcholine receptors (anti-AChRs), in which thymic pathology plays a central pathogenic role. While thymomas predominantly arise in the anterior mediastinum, ectopic locations represent a rare and clinically distinct entity. Among these, preaortic ectopic thymomas are exceedingly rare, and their association with refractory MG has not previously been described.

We report a 48-year-old woman with generalized MG with bulbar predominance, presenting with an eight-month history, confirmed by markedly elevated anti-AChR antibodies (91.1 nmol/L), a positive neostigmine test, and a pathological decrement exceeding 10% on repetitive nerve stimulation across three nerve-muscle pairs. The patient experienced a life-threatening myasthenic crisis requiring ICU admission and failed to achieve sustained remission despite corticosteroids, pyridostigmine, intravenous immunoglobulins (IVIg), and two cycles of rituximab (1000 mg at day 1 and day 15, followed by 1000 mg at six months), fulfilling criteria for refractory MG. After repeated immunotherapy failures and a normal chest X-ray, a prior external CT, initially interpreted as normal or possibly representing ectopic thyroid tissue, was followed by a dedicated thoracic CT at our institution, which identified a left-lateralized anterior mediastinal soft-tissue mass in close preaortic contact with the ascending aorta (42 × 28 × 41 mm). Complete surgical resection via median sternotomy was performed. Histopathological investigation confirmed a WHO type B2 thymoma (2021 WHO classification), with identification of Hassall's corpuscles confirming the thymic origin of the mass. The patient achieved complete clinical remission (Besinger score 100/100; MGC score 0/50) following postoperative IVIg and was subsequently tapered off corticosteroids.

This case highlights the necessity of systematic thoracic CT in all refractory MG regardless of plain radiograph findings and prior imaging reports and underscores the potential importance of complete surgical excision of preaortic ectopic thymic tissue in achieving disease control. Following a systematic search of PubMed, Scopus, and Google Scholar, we did not identify any previously reported case of a WHO type B2 preaortic ectopic thymoma described as the underlying cause of refractory generalized MG.

## Introduction

Myasthenia gravis (MG) is a chronic autoimmune neuromuscular disorder characterized by fluctuating weakness and fatigability of skeletal muscles, resulting from impaired synaptic transmission at the neuromuscular junction, primarily mediated by autoantibodies directed against acetylcholine receptors (anti-AChRs). The thymus is central to the pathogenesis of MG. Thymic hyperplasia is seen in about 60-70% of patients [1], and thymoma in 10-15% of cases, with symptomatic MG developing in 30-50% of patients with thymoma during the course of their disease [1,2].

Thymomas arise predominantly in the anterior mediastinum, but ectopic thymomas, defined as thymomas arising outside the boundaries of the normal midline pre-vascular thymic lobe, represent less than 4% of all thymic epithelial neoplasms [3,4]. The thymus originates embryologically from the third and fourth pharyngeal pouches and descends along a defined pathway from the angle of the mandible to the anterior mediastinum; failure of complete descent or aberrant migration along this route may leave ectopic thymic rests at various sites, most commonly the cervical region, intrapulmonary parenchyma, pleura, and thyroid gland [3,4]. Ectopic thymic tissue preserves its immunogenic capacity and can independently sustain anti-AChR autoantibody production, contributing to MG persistence despite standard treatment [5]. Preaortic and aortopulmonary window locations are substantially rarer still, with fewer than 10 such cases reported in the published literature, the majority without associated MG [6,7]. Their left-lateralized position outside the normal midline thymic lobe and intimate contact with the ascending aorta may lead to misidentification as lymphadenopathy, paraganglioma, or ectopic thyroid tissue on initial imaging, as illustrated by our case, resulting in diagnostic delay [8].

WHO type B2 thymomas, defined by neoplastic epithelial cells with vesicular nuclei set within a dense immature T-lymphocyte background, carry the strongest association with MG among all thymoma subtypes and are disproportionately represented in severe MGFA stage IV-V presentations and treatment-refractory disease [1]. The combination of a B2 histotype and a preaortic ectopic location thus represents a dual layer of clinical severity and topographic exceptionality. MG is defined as refractory when corticosteroids and at least one conventional immunosuppressive agent fail to achieve disease control or prevent recurrent myasthenic crises, a condition significantly more common in thymomatous forms [2].

We report this case for three specific educational purposes: (1) to illustrate the diagnostic pitfalls associated with preaortic ectopic thymomas, including the failure of both plain radiography and initial cross-sectional imaging to identify the lesion; (2) to describe a case in which complete surgical resection of an occult preaortic thymoma was followed by clinical remission of refractory MG after failure of multiple immunotherapy lines; and (3) to provide a radiological, surgical, and histopathological description of this rare topographic variant that may help guide clinicians encountering similar presentations. Following a systematic search of the published literature, we did not identify any previously reported similar case combining a preaortic ectopic location with WHO type B2 histotype and refractory generalized MG.

## Case presentation

A 48-year-old woman with no prior medical history presented with an eight-month history of fluctuating bulbo-ocular symptoms, including dysphagia with aspiration, nasal voice, binocular diplopia, and bilateral ptosis, in the absence of systemic constitutional signs. Symptoms were characteristically worsened by sustained activity and partially relieved by rest, consistent with fatigable weakness of neuromuscular origin.

Diagnostic workup

Initial investigations

The markedly elevated anti-AChR antibody titer (182-fold above the upper limit of normal) was strongly consistent with autoimmune MG and, in retrospect, indicative of an underlying thymoma-associated immunological drive (Table 1).

**Table 1:** Initial investigation results.

Investigation	Result
Prostigmine (neostigmine) test	Positive
Anti-AChR antibodies	91.1 nmol/L (N < 0.5)
Anti-MuSK antibodies	Negative
Chest X-ray	No abnormality detected

Electrophysiological Findings

Repetitive nerve stimulation (RNS) at 3 Hz and needle electromyography were performed to characterize the neuromuscular junction (NMJ) dysfunction (Table 2).

**Table 2:** Repetitive nerve stimulation results. Repetitive nerve stimulation at 3 Hz demonstrated pathological decremental responses (>10%) in the right anconeus muscle (16.9%) and left trapezius muscle (13.9%), indicating a postsynaptic neuromuscular transmission defect. Additional muscles, including the orbicularis oculi and abductor digiti minimi bilaterally, showed post-exercise facilitation without significant decrement. Lower-limb recordings were normal. These findings are consistent with generalized autoimmune myasthenia gravis.

Side	Time	Freq., Hz	Stim nmbr	Baseline Amplitude (mV)	Decrement Amplitude % (1→5)	Decrement Amplitude % (1→last)	Baseline Surface (mV×ms)	Decrement Surface %(1→5)	Post-exercise Response
Hypoglossal — Floor of Mouth									
R	00:00	3.0	10	5.16	6.7	7.4	11.8	11.5	Normal
Orbicularis Oculi — Facial Nerve R									
R	00:00	3.0	10	2.15	+9.4	+7.2	9.4	8.4	Facilitation
Orbicularis Oculi — Facial Nerve L									
L	00:00	3.0	10	3.1	+6.7	+5.8	10.8	+7.7	Facilitation
Trapezius — Spinal Accessory C2C3C4									
R	00:00	3.0	10	11.9	9.9	13.9	86.9	37.7	Exhaustion
L	00:00	3.0	10	8.26	2.6	12.9	48.7	14.8	Exhaustion
Radial–Anconeus C7 R									
R	00:00	3.0	10	5.34	16.9	10.3	7.9	0.30	Facilitation
	05:16	3.0	10	4.57	13.7	7.6	6.9	3.4	
Radial–Anconeus C7 L									
L	00:00	3.0	10	4.99	10.3	5.4	9.5	12.4	Facilitation
	01:12	3.0	10	7.53	7.7	5.1	14.6	3.8	
	03:27	3.0	10	5.05	13.7	5.6	10.1	15.4	
Abductor Digiti Minimi — Ulnar C8T1 R									
R	00:00	3.0	10	11.7	2.9	+1.6	37.6	6.6	Facilitation
	02:56	3.0	10	12.9	7.2	3.8	48.1	16.4	
	03:58	3.0	10	12.2	2.0	1.1	43.5	8.3	
	04:57	3.0	10	12.8	2.6	2.2	47.2	12.4	
Abductor Digiti Minimi — Ulnar C8T1 L									
L	00:00	3.0	10	6.8	+1.3	+9.3	19.4	5.2	Facilitation
	01:24	3.0	10	7.07	0.8	+2.7	23.1	4.3	
	02:10	3.0	10	5.53	0.09	+2.3	12.2	21.5	
	02:33	3.0	10	5.62	1.6	+4.1	12.6	26.6	
	03:32	3.0	10	7.3	28.2	18.9	26.9	64.5	
	03:47	3.0	10	7.47	26.9	22.0	26.6	65.2	
Extensor Digitorum — Fibular L4L5S1 R									
R	00:00	3.0	10	3.83	+1.3	+2.4	12.7	2.8	Facilitation
	00:30	3.0	10	3.92	+0.19	+0.9	12.5	3.4	
Extensor Digitorum — Fibular L4L5S1 L									
L	00:00	3.0	10	2.76	+1.7	+3.6	9.3	2.0	Facilitation
	00:26	3.0	10	2.85	+1.4	+2.8	9.3	1.1	

The combination of markedly elevated anti-AChR antibodies (91.1 nmol/L), a positive neostigmine test, and a pathological decrement exceeding 10% on low-frequency RNS (3 Hz) across multiple nerve-muscle pairs, with characteristic post-exercise facilitation followed by exhaustion, fulfills the diagnostic criteria for definite autoimmune myasthenia gravis as established by the MGFA Task Force [9]. RNS findings are considered supportive of the diagnosis in the context of this multimodal evaluation and are not interpreted in isolation. The involvement of the trapezius-spinal accessory pair is particularly relevant given the prominent bulbar phenotype.

Initial Treatment and Clinical Course

Prednisone was initiated at 1 mg/kg/day with potassium and calcium supplementation, combined with pyridostigmine 80 mg three times daily. No clinically significant improvement was observed (Besinger score [10]: 60/100; equivalent MGC score [11]: approximately 25/50, indicating moderate-to-severe functional impairment).

The patient subsequently developed an acute myasthenic crisis with severe respiratory failure: SpO₂ 52% on 10 L/min O₂, cyanosis, marked worsening of dysphagia with aspiration, and altered consciousness (Besinger score nadir: 20/100; equivalent MGC score: approximately 42/50, indicating severe life-threatening deficit). She was transferred to the intensive care unit, where IVIg was administered at 0.4 g/kg/day for five consecutive days, resulting in partial clinical improvement (Besinger score: 60/100; equivalent MGC score: approximately 25/50).

Persistent aspiration and incomplete remission despite IVIg prompted two cycles of rituximab (anti-CD20 monoclonal antibody), administered using the standard high-dose protocol: rituximab 1000 mg intravenously at day 1 and day 15 (first cycle), followed by a single infusion of rituximab 1000 mg six months later (second cycle). Standard pre-medication was administered prior to each infusion. Despite adequate immunosuppression, no clinically satisfactory or sustained response was observed after either cycle, defining a refractory form of MG according to established criteria - failure of corticosteroids and at least one rescue immunotherapy to achieve sustained disease control [2].

Imaging: Thymoma Identification

It is noteworthy that contrast-enhanced thoracic CT had been performed externally prior to admission, but was interpreted either as normal or as potentially representing ectopic thyroid tissue, a misinterpretation that led to a significant diagnostic delay of several months. In view of persistent MG refractory to multiple immunotherapies, a repeat dedicated thoracic CT was performed at our institution specifically to investigate a possible ectopic thymic source. This CT identified a left-lateralized anterior mediastinal soft-tissue mass extending posteriorly in close contact with the ascending aorta, with loss of the fat cleavage plane, measuring 42 × 28 × 41 mm. The lesion showed spontaneous isodensity, peripheral calcifications, and mild enhancement after contrast injection - raising strong suspicion for a preaortic ectopic thymoma. The non-enhanced CT series is presented (Figures 1A, 1B); the contrast-enhanced series could not be retrieved from the institutional PACS due to a technical archiving issue, though enhancement characteristics are formally documented in the written radiology report. Standard chest X-ray, performed prior to admission, had been entirely normal, underscoring its inadequacy for detecting ectopic thymic lesions and confirming that a normal radiograph cannot exclude an underlying thymoma [8].

**Figure 1:**
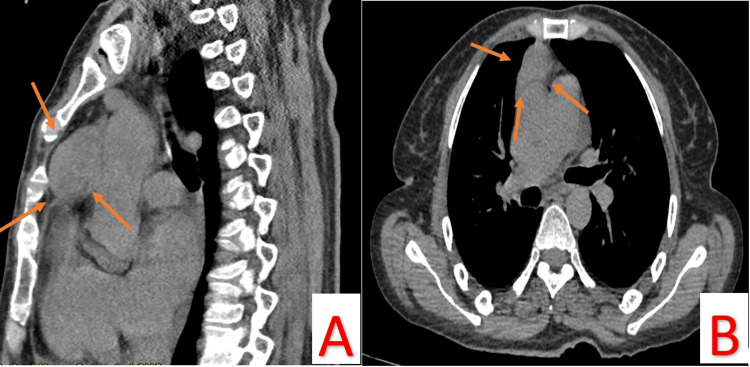
Thoracic CT: (A) sagittal view and (B) axial view demonstrating a left-lateralized anterior mediastinal soft-tissue mass (42 × 28 × 41 mm), spontaneously isodense with peripheral calcifications, extending posteriorly into close contact with the ascending aorta with loss of the fat cleavage plane, consistent with a preaortic ectopic thymoma.

Surgical Resection: Thymectomy

The patient was referred to the Department of Thoracic Surgery, where complete surgical resection was performed via median sternotomy. The transsternal approach was selected on the basis of the specific topographic characteristics of the lesion: a left-lateralized preaortic mass in intimate contact with the ascending aorta with documented loss of the fat cleavage plane, requiring wide surgical exposure of the aortopulmonary window for safe vascular dissection and direct aortic control. In the context of refractory MG, extended thymectomy with complete mediastinal fatty tissue clearance was additionally performed to eliminate potential residual ectopic thymic rests.

An encapsulated soft-tissue mass weighing 28.7 g and measuring 7 × 4.5 × 1.5 cm was excised en bloc. The procedure was uneventful, with no intraoperative vascular complications. Postoperative IVIg was administered prophylactically. No myasthenic decompensation or crisis occurred in the postoperative period. The patient was monitored in the thoracic surgery department for seven days before transfer to the neurology department.

Pathological Findings

Macroscopic examination: The surgical specimen consisted of a fibroadipose tissue fragment weighing 28.7 g and measuring 7 × 4.5 × 1.5 cm (Figure 2). Cross-sections showed a well-demarcated encapsulated mass 4 x 3.5 x 3.5 cm. The capsule was focally thickened. Areas of cystic and haemorrhagic change were seen. The nearest surgical margin was 0.1 cm.

**Figure 2:**
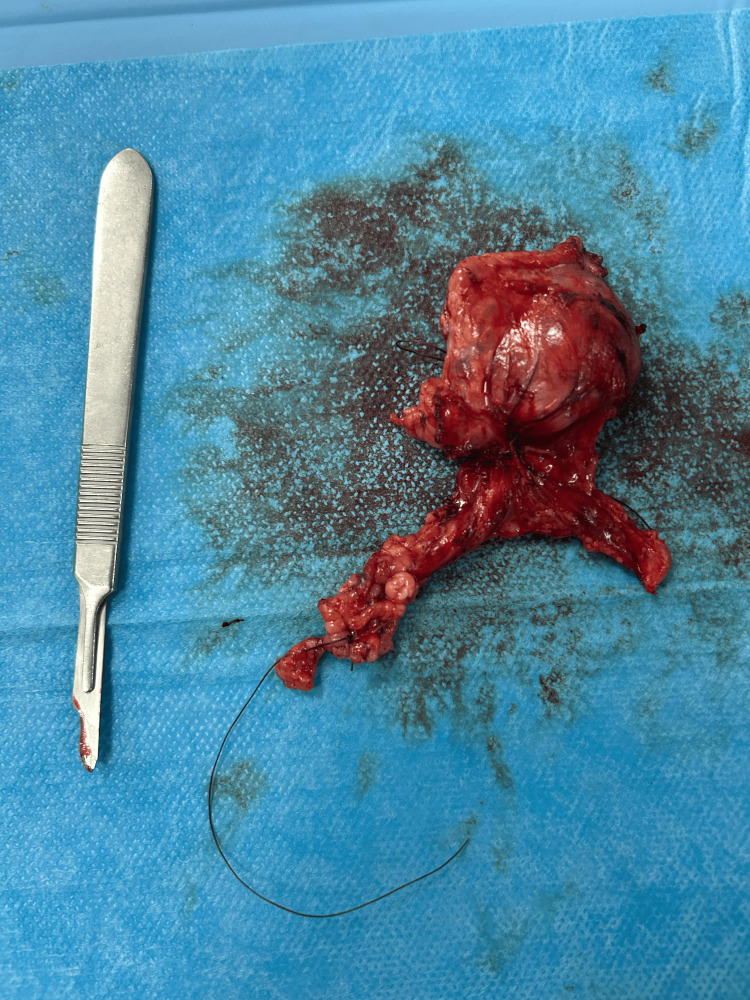
Macroscopic appearance of the resected preaortic thymoma (7 × 4.5 × 1.5 cm), showing a well-encapsulated, lobulated soft-tissue mass with areas of cystic change on cross-section.

Microscopic examination: Histological sections showed a well-demarcated encapsulated tumor arranged in lobules within a fibroadipose stroma (Figure 3). Two distinct cell populations were identified: (1) large polygonal epithelial cells with pale, rounded, occasionally grooved nuclei and prominent nucleoli, with weakly eosinophilic cytoplasm, and no atypical mitoses; (2) small lymphoid cells with hyperchromatic, regular nuclei and no atypical mitoses. Prominent perivascular spaces with eosinophilic content were present throughout, with focal capsular thickening without definitive capsular invasion. Hassall's corpuscles were identified within the interstitial fibrous stroma, confirming the thymic epithelial origin of the neoplasm and supporting its classification as arising from ectopic thymic tissue independent of topographic location. No tumor necrosis was identified. The closest surgical margin measured 0.1 cm.

**Figure 3:**
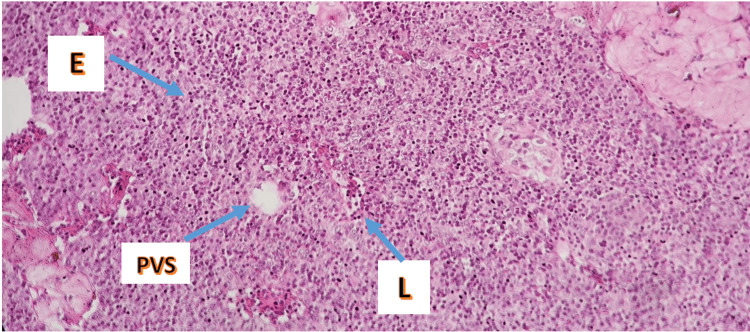
Histopathology (H&E stain, ×200). WHO type B2 thymoma. (E) Large neoplastic epithelial cells with pale vesicular nuclei and prominent nucleoli; (L) small immature lymphocytes with hyperchromatic nuclei forming a dense background; (PVS) prominent perivascular spaces, characteristic features of B2 thymoma. No necrosis or atypical mitoses identified.

Immunohistochemical analysis was performed using automated immunoperoxidase staining (OMNIS station, Agilent) to confirm the histological diagnosis and characterize the two cell populations. The results are shown in Table 3.

**Table 3:** Immunohistochemistry (automated immunoperoxidase, Agilent OMNIS platform).

Marker	Result	Interpretation
P63 (4A-4)	Positive	Moderate nuclear expression in epithelial cells — confirms epithelial lineage
CD1a	Strongly positive	Intense membranous expression in immature T lymphocytes — hallmark of B2
CD117 (c-Kit)	Negative	Excludes seminoma, GIST, and other CD117+ mediastinal tumors

Final histopathological diagnosis was WHO type B2 thymoma (2021 classification) [12]; 4 cm maximum diameter; closest surgical margin 0.1 cm; no histological features of malignancy.

Postoperative Outcome and Follow-Up

Following complete surgical resection, the patient was monitored in the thoracic surgery department for seven days postoperatively without complication, then transferred to the neurology department. Neurological follow-up was conducted at one month, three months, and six months post-resection at the neurology outpatient clinic.

At the one-month follow-up visit, significant clinical improvement was documented with regression of bulbar symptoms. A third cycle of rituximab was administered at month two post-resection as part of the planned immunotherapy protocol. At the three-month visit, corticosteroids were being actively tapered. At the six-month follow-up - the most recent visit at the time of manuscript preparation - the patient had achieved complete clinical remission (Besinger score 100/100; MGC score 0/50; equivalent MG-ADL score 0/24), corticosteroids had been successfully discontinued, and no new neurological episode had occurred.

Oncological surveillance with contrast-enhanced thoracic CT is planned at 12 months post-resection, given the WHO type B2 histotype and the close surgical margin of 0.1 cm, in accordance with standard follow-up recommendations for thymoma.

The complete clinical remission observed after surgical resection, following failure of corticosteroids, IVIg, and two cycles of rituximab, is suggestive of a causal contribution of the preaortic ectopic thymic mass to the refractory autoimmune process, although a causal relationship cannot be definitively established from a single case.

## Discussion

The thymus in MG pathogenesis: immunogenic potential of ectopic tissue

The thymus is the central orchestrator of MG pathogenesis, functioning as the primary site for T-cell education and the maintenance of self-tolerance. In the context of MG, thymic dysfunction, whether through hyperplasia or neoplastic transformation, disrupts central tolerance mechanisms and triggers the generation of autoreactive T cells that drive anti-AChR antibody production by B cells [1]. This immunological cascade is not restricted to orthotopic thymic tissue: ectopic thymic remnants arising from aberrant embryonic migration retain the same immunogenic architecture and molecular machinery as the native thymus, including aberrant expression of NMJ-related autoantigens by medullary thymic epithelial cells [13]. Surgical removal of ectopic thymic foci has been shown to produce clinically meaningful improvement in MG symptoms, directly implicating these aberrant tissues in the perpetuation of autoimmunity [5]. Critically, studies of patients undergoing re-thymectomy for refractory MG identify residual thymic tissue or ectopic foci in up to 81% of cases, a striking figure that underscores the imperative of exhaustive thymic mapping before concluding that medical refractoriness is irreversible [5].

Preaortic ectopic thymoma: topographic rarity and diagnostic challenges

As outlined in the Introduction, preaortic and aortopulmonary window thymomas are exceptionally rare, with intrapericardial thymomas representing a related but topographically distinct subtype within the broader spectrum of ectopic thymic neoplasia [7]. This rarity generates substantial diagnostic challenges: preaortic masses are frequently misidentified on imaging as mediastinal lymphadenopathy, paragangliomas, or primary vascular tumors, leading to diagnostic delay [3,6,7]. A further pitfall illustrated by our case is that preaortic thymomas may be misidentified even on cross-sectional imaging: an external CT had previously suggested either a normal study or possible ectopic thyroid tissue, a misinterpretation that delayed the correct diagnosis by several months and underscores that interpretation should ideally occur in a multidisciplinary setting when clinical suspicion is high. Standard chest radiography is similarly unreliable, as illustrated by the entirely normal plain chest X-ray in our patient, confirming that a normal radiograph cannot exclude an ectopic thymic lesion [8]. Contrast-enhanced thoracic CT remains the reference standard for detecting and characterizing these atypical locations [8,14].

The ectopic classification in our case is supported by three converging arguments: (1) the left-lateralized position of the lesion on CT, inconsistent with orthotopic thymic tissue, which is characteristically located in the midline pre-vascular space; (2) the loss of the fat cleavage plane between the mass and the ascending aorta, placing it at the interface between the anterior mediastinum and the aortopulmonary window, outside the confines of the standard thymic lobe; and (3) the histopathological identification of Hassall's corpuscles in a mass that was not contiguous with the orthotopic thymic lobe at surgical exploration, with the surgical team confirming that a distinct dissection plane was required.

The normal chest X-ray had failed to raise suspicion, a prior external CT had been misinterpreted, and CT was only performed at our institution after failure of multiple lines of immunotherapy. This sequence suggests that contrast-enhanced thoracic CT may be of value early in the workup of generalized MG that fails to respond to initial treatment, particularly when prior imaging has been inconclusive.

WHO type B2 histotype - prognostic significance in thymoma-associated MG

The WHO histological classification of thymoma carries major prognostic implications in MG-associated disease. Type B2 thymomas, defined by the predominance of large neoplastic epithelial cells with vesicular nuclei and prominent nucleoli set within a dense background of immature CD1a-positive T lymphocytes, exhibit the strongest statistical association with MG among all thymoma subtypes, significantly exceeding the MG prevalence seen with types A or AB [1]. This biological correlation is mechanistically grounded: the dense immature T-lymphocyte population characteristic of B2 thymomas reflects a failure of central tolerance induction, generating autoreactive T cells that escape thymic deletion and drive peripheral anti-AChR antibody production [1].

At the clinical level, the B2 histotype is consistently associated with more severe MG presentations. Multiple case series and cohort studies report that B2 and B3 thymomas disproportionately underlie MGFA stage IV-V disease [9], a higher incidence of myasthenic crisis, lower complete remission rates following thymectomy, and a significantly higher proportion of treatment-refractory forms [2,15-19]. Álvarez-Velasco et al., in a large multicenter cohort of thymoma-associated MG, confirmed that thymomatous patients had more generalized disease, more frequent crises, and poorer long-term outcomes [18]. It should be acknowledged that definitive evidence establishing B2 histotype as an independent predictor of refractoriness remains primarily derived from case reports and small retrospective series, and no large prospective study has yet confirmed this relationship [1,20].

Our patient illustrates the unfavorable phenotype associated with B2 histotype: markedly elevated anti-AChR titers (91.1 nmol/L), bulbar-predominant generalized MG, a life-threatening myasthenic crisis requiring ICU admission (Besinger score nadir 20/100; MGC ~42/50), and failure of two lines of immunotherapy prior to surgical identification of the thymoma. The IHC profile, strong CD1a positivity in immature lymphocytes, moderate P63 nuclear expression in epithelial cells, CD117 negativity, and the presence of Hassall's corpuscles, was fully consistent with WHO 2021 type B2 classification.

Refractory MG: therapeutic escalation and the decisive role of complete surgical resection

MG is classified as refractory when corticosteroids and at least one conventional first-line immunosuppressive agent fail to achieve sustained disease control or prevent recurrent myasthenic crises [2]. In the thymomatous MG series, refractory rates and disease-related mortality are significantly higher than in non-thymomatous forms [16].

Rescue therapies including IVIg and plasma exchange provide important short-term benefits during myasthenic crisis but do not address the underlying immunogenic source [15]. Among targeted biotherapies, rituximab has demonstrated efficacy in refractory anti-AChR-positive MG, though responses are less consistent than in anti-MuSK MG [2]. Eculizumab, a terminal complement inhibitor, has shown robust evidence in refractory generalized MG: Jin et al. reported that eculizumab effectively controlled symptoms in thymomatous refractory MG with a favorable safety profile [20]. Belimumab and other emerging agents remain investigational [16].

Complete surgical resection of the thymoma, including ectopic foci, remains the cornerstone of treatment for both oncological control and MG stabilization [1,6,18,19]. For advanced B2 thymomas or those with close surgical margins, current guidelines recommend a multimodal approach combining surgery with adjuvant radiotherapy and, in selected cases, platinum-based chemotherapy [17].

In our patient, complete clinical remission (Besinger 100/100; MGC 0/50) was observed only after surgical resection, following prior failure of corticosteroids, IVIg, and two cycles of rituximab. This sequence is suggestive of the preaortic ectopic mass acting as an immunological driver sustaining the refractory state, although this inference is necessarily limited by the single-case design. The close surgical margin (0.1 cm) warrants careful long-term oncological surveillance, with consideration of adjuvant radiotherapy per multidisciplinary tumor board assessment.

Limitations

Several limitations deserve acknowledgment. First, as with all case reports, causal inferences must be interpreted with caution. Second, the contrast-enhanced CT series could not be retrieved from the institutional PACS due to a technical archiving issue; enhancement characteristics are however documented in the written radiology report. Third, serial anti-AChR antibody titers were not reported postoperatively. Fourth, single-fiber EMG was not performed. Fifth, the closest resection margin of 0.1 cm warrants formal multidisciplinary review regarding adjuvant radiotherapy. Finally, clinicians should be aware that de novo MG may rarely appear after thymectomy for a non-myasthenic thymoma, so-called post-thymectomy MG, reflecting the persistence of autoreactive peripheral T-cell clones generated by the tumor prior to resection [7]. Long-term neurological surveillance is therefore warranted in all patients after thymectomy.

## Conclusions

This case carries three directly actionable clinical messages. First, a normal chest X-ray and even a previously reported normal or inconclusive CT do not exclude an underlying thymoma in a patient with refractory generalized MG; contrast-enhanced thoracic CT performed in a specialized multidisciplinary setting is mandatory. Second, preaortic and aortopulmonary window locations must be systematically included in the radiological differential diagnosis of anterior mediastinal masses in the context of MG, as their atypical position outside the normal midline thymic lobe may lead to misidentification as lymphadenopathy, paraganglioma, or ectopic thyroid tissue, resulting in significant diagnostic delay. Third, complete surgical resection of an occult preaortic ectopic thymoma, even after failure of multiple immunotherapy lines including rituximab, can achieve complete and durable clinical remission (Besinger 100/100; MGC 0/50), confirming the irreplaceable role of surgery in thymomatous refractory MG. These messages are directly applicable to any neurologist, thoracic surgeon, or radiologist encountering a patient with treatment-resistant MG.
